# The group IV-A cyclic nucleotide-gated channels, CNGC19 and CNGC20, localize to the vacuole membrane in *Arabidopsis thaliana*

**DOI:** 10.1093/aobpla/plt012

**Published:** 2013-02-22

**Authors:** Christen C. Y. Yuen, David A. Christopher

**Affiliations:** Department of Molecular Biosciences and Bioengineering, University of Hawaii, 1955 East-West Road, Honolulu, HI 96822, USA

**Keywords:** Calcium signalling, cation channels, cyclic nucleotide, secretory pathway, vacuole

## Abstract

Plant cyclic nucleotide-gated channels (CNGCs) are implicated in the uptake of both essential and toxic cations, Ca^2+^ signalling, and responses to biotic and abiotic stress. The 20 CNGC paralogues of *Arabidopsis* are divided into five evolutionary groups. Group IV-A is highly isolated and consists only of two closely spaced genes, *CNGC19* and *CNGC20*. Prior studies have shown that both genes are induced by salinity and biotic stress. A unique feature of CNGC19 and CNGC20 is their long hydrophilic N-termini. To determine the subcellular locations of CNGC19 and CNGC20, partial and full-length fusions to GFP(S65T) were generated. Translational fusions of the N-termini of CNGC19 (residues 1–171) and CNGC20 (residues 1–200) to GFP(S65T) were targeted to punctate structures when transiently expressed in leaf protoplasts. In the case of CNGC20, but not CNGC19, the punctate structures were co-labelled with a marker for the Golgi. The full-length CNGC19-GFP fusion co-localized with markers for the vacuole membrane (αTIP- and γTIP-mCherry). Vacuole membrane labelling by the full-length CNGC20-GFP fusion was also observed, but the signal was weak and accompanied by numerous punctate signals that did not co-localize with αTIP- or γTIP-mCherry. These punctate structures diminished, and localization of full-length CNGC20-GFP to the vacuole increased, when it was co-expressed with the full-length CNGC19-mCherry. Vacuole membrane labelling was also detected *in planta* via immunoelectron microscopy using a CNGC20-antiserum on cryopreserved ultrathin sections of roots. We hypothesize that the role of group IV-A CNGCs is to mediate the movement of cations between the central vacuole and the cytosol in response to certain types of abiotic and biotic stress.

## Abstract (precis)

The cyclic nucleotide-gated channels, CNGC19 and CNGC20, are the sole members of the highly isolated evolutionary group IV-A in Arabidopsis plants. Prior studies have shown that the expression of both CNGC19 and CNGC20 genes are induced by salinity and biotic stress. In this report, CNGC19 and CNGC20 were determined to localize to the vacuolar membrane. Co-expression of CNGC19 and CNGC20 increased the efficiency of vacuolar localization. CNGC19 and CNGC20 are, therefore, vacuolar membrane channels that are hypothesized to mediate plant response to salinity and biotic stress.

## Introduction

Plants have evolved several distinct classes of transporters and channels to facilitate the movement of cations across cellular membranes (Mäser *et al.* 2001). The members of the plant cyclic nucleotide-gated channel (CNGC) family are evolutionarily and structurally related to Shaker-type K^+^ channels, but are typically permeable to a range of monovalent cations (Leng *et al.* 2002; Balagué *et al.* 2003; Hua *et al.* 2003; Gobert *et al.* 2006; Christopher *et al.* 2007). Several CNGCs have also been shown to translocate divalent cations such as Ca^2+^ and Mg^2+^ (Leng *et al.* 1999; Ali *et al.* 2006; Frietsch *et al.* 2007; Urquhart *et al.* 2007; Guo *et al.* 2010), and there is a growing body of evidence that the molecular function of some CNGCs is to mediate Ca^2+^ signalling (Talke *et al.* 2003; Ma *et al.* 2009). Cyclic nucleotide-gated channels are oppositely regulated by two distinct second messengers, cyclic nucleotides and Ca^2+^/calmodulin, through partially overlapping ligand-binding domains located near the C-terminus (Leng *et al.* 1999, 2002; Arazi *et al.* 2000; Köhler and Neuhaus 2000; Balagué *et al.* 2003; Li *et al.* 2005; Yoshioka *et al.* 2006). In general, cyclic nucleotides activate CNGCs, whereas Ca^2+^/calmodulin inhibit them. The phenotypic characterization of *cngc* mutants and antisense lines implicates members of this channel family in the uptake and distribution of monovalent and divalent cations (Sunkar *et al.* 2000; Li *et al.* 2005; Gobert *et al.* 2006; Ma *et al.* 2006; Christopher *et al.* 2007; Guo *et al.* 2008, 2010; Yuen and Christopher 2010), plant defence responses (Yu *et al.* 1998; Clough *et al.* 2000; Balagué *et al.* 2003; Yoshioka *et al.* 2006; Moeder *et al.* 2011), gravitropism (Ma *et al.* 2006; Borsics *et al.* 2007) and pollen tube elongation (Chang *et al.* 2007; Frietsch *et al.* 2007).

Plant CNGCs are divided into five phylogenetic subfamilies, designated as groups I, II, III, IV-A and IV-B (Mäser *et al.* 2001). Of the 20 CNGCs in *Arabidopsis*, only two genes, *CNGC19* and *CNGC20*, constitute group IV-A. These tandem genes are closely spaced on chromosome 3. Whereas *CNGC19* is expressed within the vasculature of roots and leaves, *CNGC20* is expressed in the root cortex, the carpel and sepals of flowers, guard cells and in leaf mesophyll cells proximal to veins (Kugler *et al.* 2009). Although salt stress influences the expression of both genes (Maathuis 2006; Kugler *et al.* 2009; Yuen and Christopher 2010), knockout mutations in either gene do not result in hypersensitivity to Na^+^, or altered accumulation of Na^+^ within plant tissues (Maathuis 2006; Kugler *et al.* 2009). Null mutants of *CNGC19* and *CNGC20* exhibit reduced resistance to avirulent pathogen infection, indicating a role for these genes in plant defence responses (Moeder *et al.* 2011). Furthermore, *CNGC19* is induced by bacterial infection and the bacterial elicitor, flg22, and T-DNA insertion mutants of *CNGC19* display increased susceptibility to the fungal pathogen, *Botrytis cinerea* (Moeder *et al.* 2011).

To understand how individual CNGCs contribute to cation fluxes in plants, it is crucial to define their distribution patterns within cells. In plants, CNGCs are present at both the plasma membrane (PM) and the membrane of vacuoles. The subcellular localization of CNGCs has primarily been accomplished through the use of chimeric fluorescent reporter proteins. Fluorescent protein fusions indicate that at least four of the six CNGCs belonging to group I are targeted to the PM: CNGC3 (Gobert *et al.* 2006), CNGC10 (Christopher *et al.* 2007) and CNGC11 and CNGC12 (Baxter *et al.* 2008). CNGC18, a pollen-specific member of group III, was shown to localize to a region of the PM near the tip of expanding pollen tubes (Chang *et al.* 2007; Frietsch *et al.* 2007). The group II members, CNGC7 and CNGC8, are also expressed exclusively in pollen, but localize to the tonoplast (Chang *et al.* 2007). The subcellular distribution of CNGCs has been studied in a few cases by immunolocalization. Using paralogue-specific antibodies, CNGC5 (group II) was detected at the PM of leaf protoplasts by immunofluorescence microscopy, and CNGC10 was shown by high-resolution immunoelectron microscopy to pass through the secretory pathway and localize to the PM (Christopher *et al.* 2007). In contrast, no experimental information is available on the subcellular localizations of CNGC19 or CNGC20. Protein sorting algorithms predict that CNGC20 resides in the chloroplast (Sherman and Fromm 2009). However, no CNGC-related sequences were identified in two comprehensive, experimentally derived datasets of the *Arabidopsis* chloroplast proteome, AT_CHLORO (Ferro *et al.* 2010) and the Plastid Proteome DataBase (van Wijk and Baginsky 2011).

In this report, we utilized fluorescent reporter protein fusions and immunolelectron microscopy to determine the subcellular destinations of CNGC19 and CNGC20. We demonstrated that both channels are targeted to vacuolar membranes. Our findings suggest that group IV-A CNGCs mediate plant responses to salinity and pathogen infection by facilitating the movement of cations between the central vacuole and the cytosol.

## Methods

### Protein sequence analysis

Subcellular localization predictions for *Arabidopsis* CNGCs were performed using TargetP v1.1 (Nielsen *et al.* 1997). Transmembrane domain predictions were performed using TMHMM v1.0 (Krogh *et al.* 2001). Genes encoding putative group IV-A CNGCs were identified from the sequenced genomes of *Oryza sativa*, *Populus trichocarpa*, and *Physcomitrella patens* by BLASTP searches, with CNGC19 and CNGC20 serving as the query sequences. The poplar homologues, designated here as PtCNGC-IVa1, PtCNGC-IVa2 and PtCNGC-IVa3 (GenBank: EEE95941.1, EEF07751.1 and EEF07752.1, respectively), and the rice homologue OsCNGC-IVa1 (GenBank: BAD16877.1) were obtained using the NCBI BLAST server, while the moss homologue PpCNGC-IVa1 (COSMOSS v1.6: Pp1s204_120V6) was identified by a Phytozome BLAST search. Phylogenetic analysis with all 20 CNGCs from *Arabidopsis* confirmed that these CNGCs belong to group IV-A (data not shown).

### Generation of translational fusion constructs

All constructs used for protoplast transfection in this study were generated in cloning vector pBluescript KS(+). The generation of pBL(35S*:GFP(S65T)*) was described previously (Cho *et al.* 2011). The constructs pBL(35S:*CNGC19*_FL_-*GFP*) and pBL(35S:*CNGC20*_FL_-*GFP*) were created by replacing the *EGFP* coding sequence of plasmid pBL(35S:*CNGC10*-*EGFP*) (Christopher *et al.* 2007) with *GFP*(*S65T*), and the *CNGC10* genomic DNA sequence with the full-length genomic DNA sequences of *CNGC19* (*At3g17690*) or *CNGC20* (*At3g17700*), respectively. The GFP(S65T) fragment was amplified from plasmid HBT95::sGFP(S65T)-NOS with primers GFP/NdeI (5′-CACCGACTAGTCATATGGTGAGCAAGGGCGAG-3′) and GFP/BstEII (5′-TACAGGGTCACCTTACTTGTACAGCTCGTCCATG-3′). *CNGC19* was amplified from *Arabidopsis thaliana* (ecotype Col-0) genomic DNA using the PCR primer pair CNGC19/NcoI.F (5′-TAAACCATGGCTCACACTAGGACTTTCACT-3′) and CNGC19/NdeI.R (5′-ATCACATATGACGGTTGGAATTGGAGTGAG-3′), while *CNGC20* was amplified using primers CNGC20/NcoI.F (5′-TGGTCAAGCCATGGCTTCCCACAACGA-3′) and CNGC20/NdeI.R (5′-AATACCATATGAAGGCTATAACTAGACTGAG-3′). The *GFP(S65T)* fragment was cloned between the NdeI and BstEII restriction sites of pBL(35S:*CNGC10*-*EGFP*), and the CNGC19 and CNGC20 fragments between NcoI and NdeI. It was necessary to ligate the *CNGC20* amplicon sequentially as two separate fragments due to the presence of an internal NdeI site. Green fluorescent protein fusions to the N-terminal regions of CNGC19 and CNGC20 were developed as described above, except that the CNGC19_N1_ fragment was obtained using the primer pair CNGC19/NcoI.F and CNGC19(D86)/NdeI.R (5′-TTGAACATATGATCATCTTCAGGTGGAAC-3′), CNGC19_N2_ using primers CNGC19/NcoI.F and CNGC19(V166)/NdeI.R (5′-GTCCACATATGAACAAATTTGGAATGAGGA-3′), CNGC20_N1_ using primers CNGC20/NcoI.F and CNGC20(E117)NdeI.R (5′-TTCAACATATGCTCATCCTCTGAGGAGTT-3′) and CNGC20_N2_ using primers CNGC20/NcoI.F and CNGC20(V200)/NdeI.R (5′-GTCCACATATGAACCTCTTTGGCATGAGGA-3′). The fusion construct pBL(35S:*CNGC19*_FL_-*mCherry*) was generated by ligation of a PCR fragment containing both the coding sequence for mCherry and the nopaline synthase (nos) 3′ untranslated region (UTR) between the NdeI and SacI sites of pBL(35S:*CNGC19*_FL_-*GFP*). This fragment was amplified from plasmid pt-rk (Nelson *et al.* 2007), using the primers GFP/NdeI.F and Nos/SacI.R (5′-TAGTTGAGCTCCCGATCTAGTAACATAGA-3).

The constructs used for the transient expression of fluorescent markers for the Golgi [pBL(35S:*Man49-mCherry*)], peroxisomes [pBL(35S:*mCherry-*SKL)] and tonoplast [pBL(35S:*γTIP-mCherry*)] are derivatives of the organelle markers developed by Nelson *et al.* (2007). The new plasmids were generated by digesting the binary plasmids G-rk, PX-rk and vac-rk (respectively) with SacI and HindIII, and mobilizing the reporter gene cassettes between the corresponding restriction sites of pBluescript KS(+). The tonoplast marker construct, pBL(35S:*αTIP*-*mCherry*), was generated by amplification of the full-length genomic DNA sequence of *AtαTIP* (*At1g73190*; also referred to as *AtTIP3;1*) with primers αTIP/XbaI.F (5′-AAGTTCTAGATCATAATGGCAACATCAGCTC-3′) and αTIP/BamHI.R (5′-GTTCGGATCCGTAATCTTCAGGGGCCAAG-3′), and subsequent ligation of the PCR product between the SpeI and BamHI sites of pBL(35S:*γTIP-mCherry*). The plastid envelope marker pBL(35S:*NTT2*-*mCherry*) was created in a similar manner, using primers NTT2/SpeI.F (5′-GTGCTACTAGTGAGATAGAGAGATGGAAGGT-3′) and NTT2/BamHI.R (5′-ATCATGGATCCAATGCCAGTAGGAGTAGATTTCT-3′) to amplify the genomic DNA sequence of *AtNTT2* (*At1g15500*). *pBL(35S:NTT2-GFP)* was created by digesting *pBL(35S:NTT2-mCherry)* with KpnI and NcoI, and cloning the 3.1-kb fragment containing the cauliflower mosaic virus (CaMV) 35S promoter and *AtNTT2* genomic DNA sequence between the KpnI and NcoI sites of *pBL(35S:GFP(S65T))*. The construct pBL(35S:*BiP1-mCherry*-HDEL) contains, in order, the CaMV 35S promoter, the *AtBiP1* (*At5g28540*) genomic DNA sequence, the coding sequence of mCherry-HDEL and the nos terminator, inserted between the KpnI and SacI sites of pBluescript KS(+). The CaMV 35S promoter region was amplified with primers 35S/KpnI.F (5′-TTCAAGGTACCTTCATGGAGTCAAAGATTCA-3′) and 35S/XhoI.R (5′-ATCTACTCGAGTCAAGAGTCCCCCGTG-3′), the *AtBiP1* gene sequence was amplified from *A. thaliana* (ecotype Col-0) genomic DNA with primers BiP1/XhoI.F (5′-GAGCTCGAGCGCAAAAGTTTCCGATATGGCTCGCTC-3′) and BiP1/XmaI.R (5′-AGACCCGGGGAGCTCATCGTGAGACTCATCTTC-3′), and the mCherry-HDEL coding sequence and nos 3′ UTR were amplified from plasmid ER-rk with primers GFP/XmaI.F (5′-AAACCCGGGCTTGTACAGCTCGTCCATGC-3′) and Nos/SacI.R.

### Transient expression in leaf protoplasts

Protoplasts were isolated from 3- to 4-week-old *Arabidopsis* leaves and transfected with plasmid DNA using the protocol described by He *et al*. (2006). For standard transfections involving only one reporter construct, 20 μL of ∼1 μg μL^−1^ plasmid DNA were added per 200 μL of protoplasts suspended in MMg solution (0.4 M mannitol, 15 mM MgCl_2_, 4 mM MES, pH 5.7). In cases where protoplasts were simultaneously co-transfected with two reporter constructs, a 20-μL mixture containing ∼10 μg of each plasmid was utilized. After transfection, the protoplasts were incubated in W5 solution (154 nM NaCl, 125 mM CaCl_2_, 5 mM KCl, 2 mM MES, pH 5.7). Samples were analysed 18+ hours post-transfection with an Olympus FV-1000 laser scanning confocal microscope. For mitochondria staining, the protoplasts were incubated in W5 solution containing 0.5 µM MitoTracker Orange CMTMRos (Life Technologies, Grand Island, NY, USA) 30 min prior to analysis. The excitation/emission filters utilized for fluorescence detection were 488/505–525 nm for GFP(S65T), 543/585–615 nm for mCherry and MitoTracker Orange, and 633/650 nm for chlorophyll autofluorescence. Confocal microscopy was performed at the Biological Electron Microscope Facility (University of Hawaii, Manoa, HI, USA).

### Immunoelectron microscopy

An affinity-purified rabbit polyclonal antibody was raised against a unique region of CNGC20 (residues 664–678, sequence Ac-CLERSSVNPDGTRIR-amide) by New England Peptide (Gardner, MA, USA). For immunogold labelling, developing roots and leaves were preserved by high-pressure freezing/freeze-substitution techniques as described previously (Andème Ondzighi *et al.* 2008). The resin-embedded sections were placed on formvar-coated nickel slot grids and blocked for 30 min with 3 % (w/v) non-fat dried milk solution in 0.01 M phosphate-buffered saline pH 7.2 containing 0.1 % Tween-20 (PBST). The sections were washed in PBST and then incubated with a 10-fold dilution of the CNGC20 primary antiserum or the rabbit pre-immune serum (negative control). After washing, a 25-fold dilution of secondary antibody, goat anti-rabbit IgG conjugated to 15 nm gold particles (Ted Pella, Inc.), was added for 2 h at room temperature. Sections were washed and stained with uranyl acetate and lead citrate. All observations were performed using a Philips CM10 microscope (Philips, Hillsboro, OR, USA). The CNGC20 antiserum was tested by immunoblot analysis **[**as shown in **Supporting Information****]** by the method described in Neuteboom *et al.* (2009).

## Results

### The hydrophilic N-terminus of group IV-A CNGCs is novel and conserved

**Figure 1.**
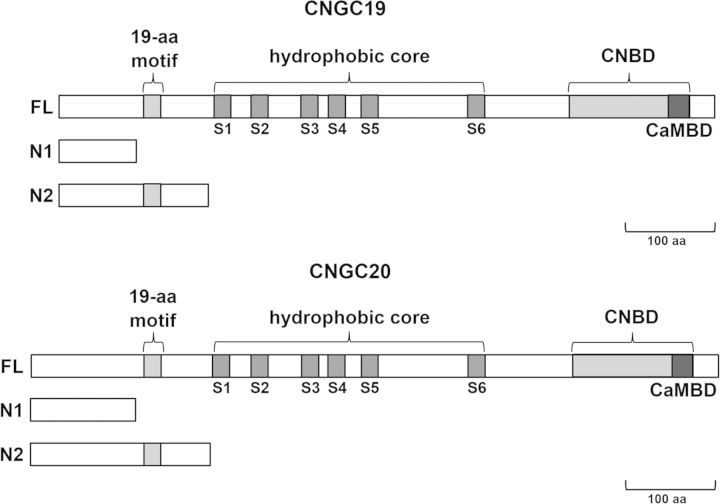
Domain arrangement of CNGC19 and CNGC20. Shaded boxes indicate the positions of the N-terminal 19-aa motif, the six transmembrane segments (S1–S6) of the hydrdophobic core region and the C-terminal overlapping cyclic nucleotide-binding (CNBD) and calmodulin-binding domains (CaMBD). FL corresponds to the full-length versions of CNGC19 and CNGC20, and N1 and N2 represent portions of the N-terminal region utilized in GFP fusion constructs.

CNGC19 and CNGC20 polypeptides possess the major structural features common to members of the plant CNGC family. These include a central hydrophobic core region consisting of six membrane-spanning α-helices (S1–S6), and partially overlapping cyclic nucleotide-binding and calmodulin-binding domains situated near their C-termini (Fig. 1). Interestingly, the N-terminal hydrophilic ends of CNGC19 and CNGC20 [171 and 205 amino acids (aa) in length, respectively] are substantially longer than the N-termini of other *Arabidopsis* CNGC paralogues (30–126 aa; Table 1). The hydrophilic N-termini of CNGC19 and CNGC20 share 59.6 % protein sequence identity, but lack significant sequence similarity to the N-termini of other *Arabidopsis* CNGCs. According to the neural network subcellular prediction program, TargetP, both CNGC19 and CNGC20 potentially harbour chloroplast localization signals (Table 1), with hypothetical transit peptide cleavage sites after positions L48 of CNGC19 and R25 of CNGC20.

**Table 1:** N-terminal length and predicted subcellular localization of *Arabidopsis* CNGCs. The 20 CNGCs of *Arabidopsis* are arranged by subfamily, with group IV-A orthologues from other plants included for comparison. Deduced N-terminal lengths are based on the predicted positions of the S1 transmembrane segments by TMHMM v1.0. Here, %19Nterm and %20Nterm indicate percentage identity with the N-terminal regions of CNGC19 and CNGC20; the absence of similarity is denoted by ‘not applicable’ (n.a.). Four predictive scores are obtained using TargetP: cTP, chloroplast transit peptide; mTP, mitochondrial targeting peptide; SP, secretory pathway signal peptide; and others. The highest score among the four categories is indicated in italics.

	Name	N-terminal region			TargetP			
		Length	%19Nterm	%20Nterm	cTP	mTP	SP	Others
I	AtCNGC1	97	n/a	n/a	0.09	0.23	0.03	*0.80*
	AtCNGC3	85	n/a	n/a	0.24	0.21	0.03	*0.35*
	AtCNGC10	81	n/a	n/a	0.11	0.28	0.02	*0.83*
	AtCNGC11	42	n/a	n/a	0.01	0.53	0.04	*0.87*
	AtCNGC12	42	n/a	n/a	0.00	0.28	0.04	*0.96*
	AtCNGC13	81	n/a	n/a	0.10	*0.74*	0.00	0.33
II	AtCNGC5	101	n/a	n/a	0.22	0.15	0.05	*0.56*
	AtCNGC6	116	n/a	n/a	0.15	0.21	0.01	*0.71*
	AtCNGC7	68	n/a	n/a	0.10	0.40	0.01	*0.70*
	AtCNGC8	104	n/a	n/a	0.21	0.33	0.01	*0.47*
	AtCNGC9	109	n/a	n/a	0.06	0.35	0.01	*0.66*
III	AtCNGC14	85	n/a	n/a	0.07	0.10	0.19	*0.93*
	AtCNGC15	81	n/a	n/a	0.02	0.31	0.08	*0.89*
	AtCNGC16	57	n/a	n/a	0.19	*0.75*	0.01	0.09
	AtCNGC17	84	n/a	n/a	0.12	0.09	0.04	*0.88*
	AtCNGC18	53	n/a	n/a	0.03	*0.51*	0.10	0.30
IV-A	AtCNGC19	171	100.0 %	59.6 %	*0.68*	0.20	0.01	0.12
	AtCNGC20	205	59.6 %	100.0 %	*0.98*	0.02	0.03	0.15
	PtCNGC-IVa1	209	39.0 %	50.2 %	0.41	0.07	0.06	*0.82*
	PtCNGC-IVa2	209	39.5 %	51.2 %	0.29	0.07	0.06	*0.85*
	PtCNGC-IVa3	196	39.4 %	46.4 %	0.19	0.08	0.05	*0.91*
	OsCNGC-IVa1	183	25.9 %	30.9 %	0.12	0.17	0.04	*0.84*
	PpCNGC-IVa1	135	22.0 %	24.9 %	0.07	0.23	0.07	*0.86*
IV-B	AtCNGC2	126	n/a	n/a	0.15	0.20	0.01	*0.52*
	AtCNGC4	92	n/a	n/a	0.10	0.33	0.09	*0.79*

**Figure 2.**
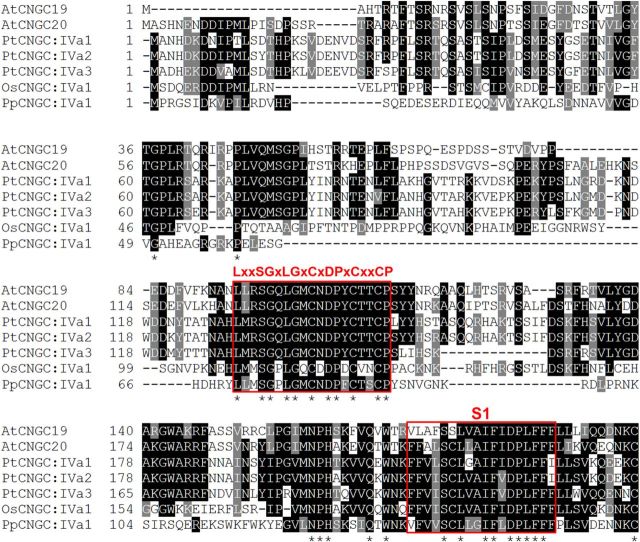
Sequence alignment of group IV-A CNGCs from *Arabidopsis*, poplar, rice and moss. Three putative members of group IV-A are present in poplar (GenBank: EEE95941.1, EEF07751.1 and EEF07752.1), one in rice (GenBank: BAD16877.1) and one in *P. patens* (COSMOSS v1.6: Pp1s204_120V6). Residues matching the consensus (five or more sequences) are shaded in black, and similar residues are shaded in grey. Amino acids strictly conserved among all sequences are denoted by an asterisk below the alignment. The 19-aa conserved interval (LxxSGxLGxCxDPxCxxCP) and first transmembrane domain (S1) are boxed in red. The indicated position of S1 is based on TMHMM 1.0 predictions for both CNGC19 and CNGC20.

We compared the N-terminal segments of CNGC19 and CNGC20 with those belonging to group IV-A CNGCs encoded by the sequenced genomes of poplar (*Populus trichocarpa*), rice (*Oryza sativa* L. ssp. *japonica*) and moss (*Physcomitrella patens* ssp. *patens*). The rice and poplar orthologues have N-terminal hydrophilic regions that are comparable in length to CNGC19 and CNGC20 (183–209 aa), while the N-terminus of the *P. patens* orthologue is substantially shorter (135 aa; Table 1). Protein sequence alignment revealed several strictly conserved residues within the hydrophilic N-terminal regions of group IV-A CNGCs, including a 19-aa interval with the conserved sequence LxxSGxLGxCxDPxCxxCP (Fig. 2). Unlike CNGC19 and CNGC20, the group IV-A CNGCs of rice, poplar and moss were not predicted to localize to chloroplasts (Table 1).

### GFP fusions with CNGC19 or CNGC20 are not targeted to chloroplasts

**Figure 3.**
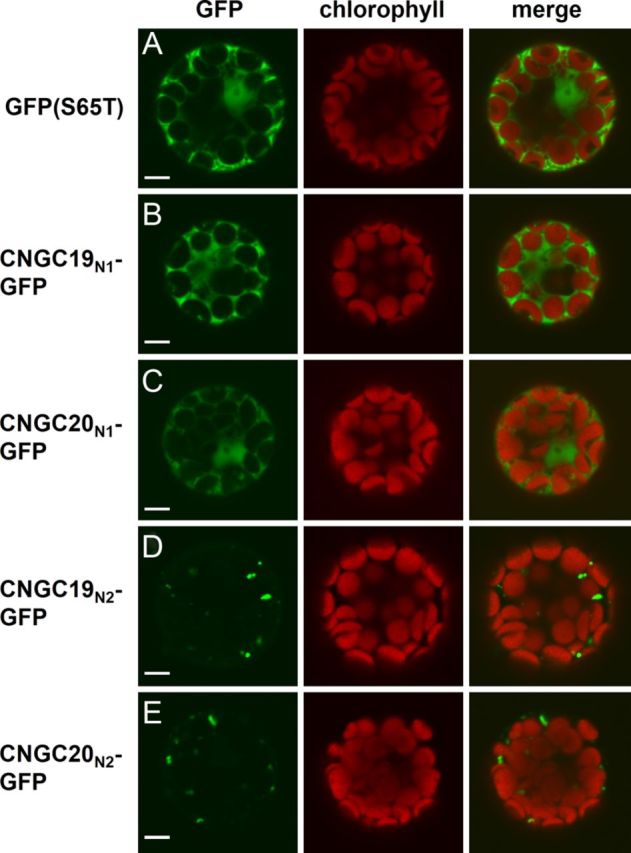
Subcellular localization pattern of GFP fused to N-terminal segments of CNGC19 or CNGC20. Confocal laser scanning microscope images of leaf protoplasts transiently expressing (A) GFP(S65T), (B) CNGC19_N1_-GFP, (C) CNGC20_N1_-GFP, (D) CNGC19_N2_-GFP or (E) CNGC20_N2_-GFP. Column 1, GFP signal (green); column 2, chlorophyll autofluorescence (red); column 3, merged GFP and chlorophyll signals. Scale bars represent 5 µm.

To determine whether CNGC19 and CNGC20 truly possess N-terminal chloroplast sorting signals, constructs were developed for the expression of the first 88 aa of CNGC19 or the first 117 aa of CNGC20, translationally fused to the N-terminus of the green fluorescent protein variant, GFP(S65T). The corresponding chimeric proteins, designated as CNGC19_N1_-GFP and CNGC20_N1_-GFP, were transiently expressed in *Arabidopsis* leaf protoplasts under the CaMV 35S promoter. Despite harbouring the predicted chloroplast transit peptide sequences of their respective CNGC paralogues (Fig. 1), expression of CNGC19_N1_-GFP and CNGC20_N1_-GFP in protoplasts resulted in diffuse cytoplasmic fluorescence (Fig. 3B and C), similar to non-chimeric GFP(S65T) controls performed in parallel (Fig. 3A). The CNGC19_N1_ and CNGC20_N1_ protein segments span from the initiator Met residue to just prior to the conserved N-terminal 19-aa motif of group IV-A CNGCs. To address the question of whether a longer interval of the N-terminus was required for chloroplast targeting, a second set of GFP fusions was generated using nearly the entire N-terminal region (CNGC19_N2_: aa 1–166; CNGC20_N2_: aa 1–200). Unlike their shorter counterparts, the longer CNGC19_N2_-GFP and CNGC20_N2_-GFP fusions displayed punctate GFP fluorescence patterns. However, these punctate signals did not overlap with chlorophyll autofluorescence (Fig. 3D and E), indicating that the CNGC19_N2_ and CNGC20_N2_ sequences directed the chimeric proteins to intracellular structures which were distinct from chloroplasts.

**Figure 4.**
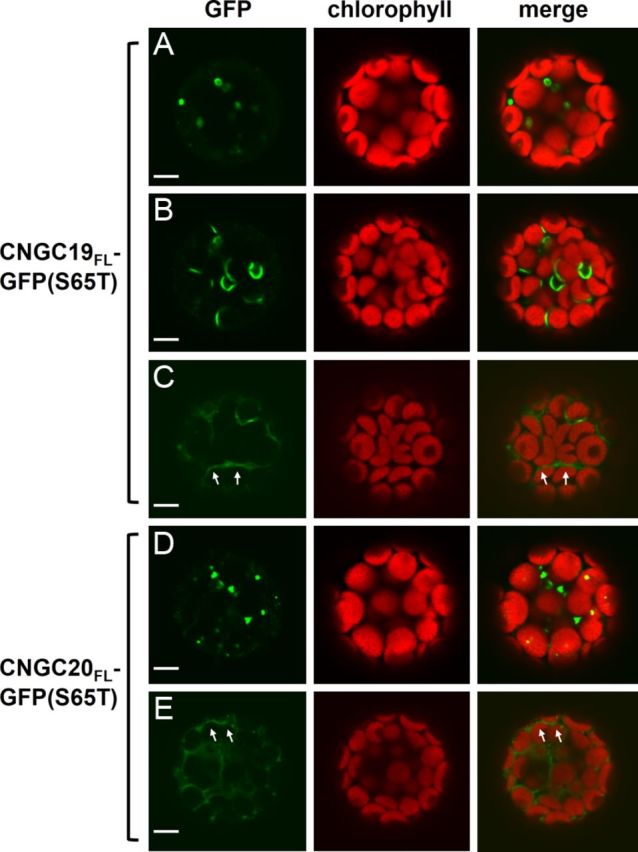
Subcellular localization pattern of GFP fused to full-length CNGC19 or CNGC20. Confocal laser scanning microscope images of leaf protoplasts transiently expressing (A–C) CNGC19_FL_-GFP or (D, E) CNGC20_FL_-GFP. The arrows indicate contiguous membranes labelled by CNGC19_FL_-GFP (C) or CNGC20_FL_-GFP (E) that wrap around multiple chloroplasts. Column 1, GFP signal (green); column 2, chlorophyll autofluorescence (red); column 3, merged GFP and chlorophyll signals. Scale bars represent 5 µm.

**Figure 5.**
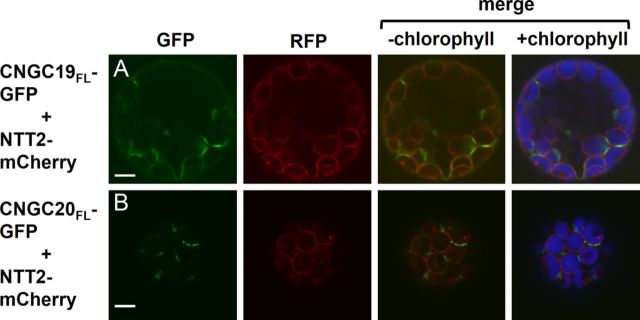
Comparison of CNGC19_FL_- and CNGC20_FL_-GFP localization with a marker for the chloroplast envelope. Confocal microscopy images of leaf protoplasts transiently expressing (A) CNGC19_FL_-GFP or (B) CNGC20_FL_-GFP, co-transfected with the chloroplast envelope marker, NTT2-GFP. Column 1, GFP signal (green); column 2, RFP signal (red); column 3, merged GFP and RFP signals; column 4, merged GFP and RFP signals with chlorophyll autofluorescence (blue). Scale bars represent 5 µm.

In addition to the truncated CNGC-GFP fusions, the localization patterns of fusions containing the full-length sequences of CNGC19 (CNGC19_FL_-GFP) or CNGC20 (CNGC20_FL_-GFP) were examined. Several distinct localization patterns were observed in protoplasts transfected with the full-length CNGC19 and CNGC20 fusion constructs. The CNGC19_FL_-GFP fusion labelled elliptical structures (Fig. 4A), short non-spherical endomembranes (Fig. 4B), as well as intracellular structures possessing an extended endomembrane morphology (Fig. 4C). The CNGC20_FL_-GFP fusion typically exhibited punctate labelling (Fig. 4D), with occasional weak labelling of extended endomembranes (Fig. 4E). The endomembrane localization patterns of CNGC19_FL_-GFP and CNGC20_FL_-GFP were not consistent with labelling of the chloroplast envelope, as contiguous endomembranes labelled by the full-length CNGC fusions were sometimes observed wrapping around two or more chloroplasts (arrows in Fig. 4C and E). A marker for the chloroplast envelope inner membrane was generated by fusing *A. thaliana* plastidic nucleotide transporter 2 (NTT2) to the N-terminus of the monomeric red fluorescent protein (RFP) variant, mCherry. Protoplasts simultaneously expressing NTT2-mCherry in combination with either CNGC19_FL_-GFP or CNGC20_FL_-GFP did not display significant co-localizing GFP and RFP fluorescence (Fig. 5). The NTT2-mCherry marker consistently labelled the perimeter of individual chloroplasts, indicating that the trafficking of proteins targeted to the chloroplast envelope was not impaired in the leaf protoplast transient expression system.

### CNGC19_FL_-GFP and CNGC20_FL_-GFP co-localize with vacuole membrane markers αTIP- and γTIP-mCherry

**Figure 6.**
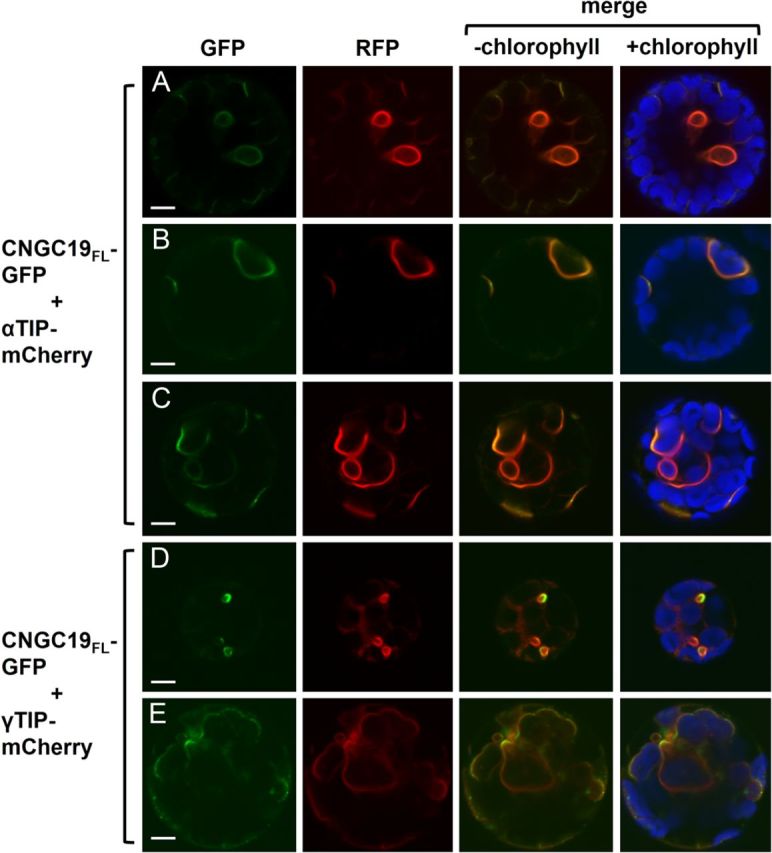
Comparison of CNGC19_FL_-GFP localization with markers for the tonoplast. Confocal laser scanning microscope images of leaf protoplasts co-transfected with CNGC19_FL_-GFP and (A–C) αTIP-mCherry or (D, E) γTIP-mCherry. Column 1, GFP signal (green); column 2, RFP signal (red); column 3, merged GFP and RFP signals; column 4, merged GFP and RFP signals with chlorophyll autofluorescence (blue). Scale bars represent 5 µm.

To address the possibility that CNGC19 and CNGC20 are targeted to vacuolar membranes, co-transfection experiments were performed with the vacuole membrane markers, α-tonoplast intrinsic protein (αTIP)-mCherry and γTIP-mCherry. Elliptical structures labelled by CNGC19_FL_-GFP were co-labelled by αTIP-mCherry (Fig. 6A) and γTIP-mCherry (Fig. 6D). The CNGC19_FL_-GFP fusion also co-localized with αTIP- and γTIP-mCherry within non-elliptical membranes (Fig. 6B, C and E). Although these membranes occasionally resembled the limiting membrane of the central vacuole (Fig. 6E), in many instances CNGC19_FL_-GFP co-localized with short regions of intense vacuole marker fluorescence (Fig. 6B and C).

**Figure 7.**
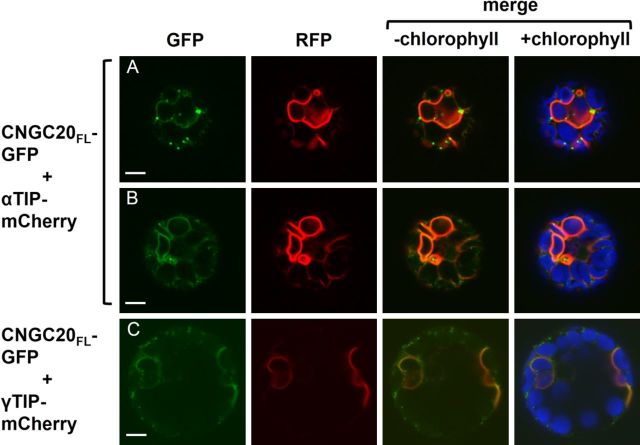
Comparison of CNGC20_FL_-GFP localization with markers for the tonoplast. Confocal laser scanning microscope images of leaf protoplasts co-transfected with CNGC20_FL_-GFP and (A, B) αTIP-mCherry or (C) γTIP-mCherry. Column 1, GFP signal (green); column 2, RFP signal (red); column 3, merged GFP and RFP signals; column 4, merged GFP and RFP signals with chlorophyll autofluorescence (blue). Scale bars represent 5 µm.

Comparison of the signal pattern of CNGC20_FL_-GFP with that of αTIP- or γTIP-mCherry revealed that CNGC20_FL_-GFP co-localized with the vacuole markers in both extended endomembranes (Fig. 7A–C) and ring-like structures (Fig. 7A and B). However, neither vacuole marker co-localized with the punctate CNGC20_FL_-GFP signals (Fig. 7A–C), implying that a substantial proportion of CNGC20_FL_-GFP fusion protein was not incorporated into vacuolar membranes.

**Figure 8.**
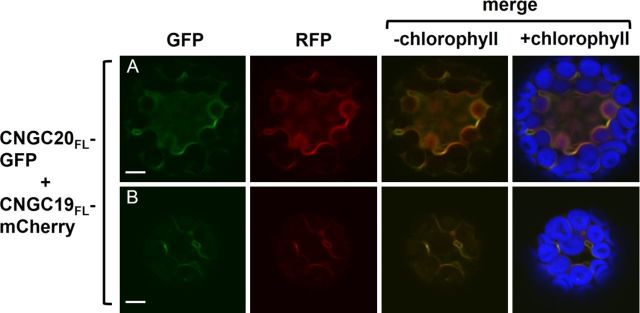
Simultaneous expression of CNGC19_FL_-mCherry and CNGC20_FL_-GFP. (A, B) Confocal laser scanning microscope images of leaf protoplasts co-transfected with CNGC19_FL_-mCherry and CNGC20_FL_-GFP. Column 1, GFP signal (green); column 2, RFP signal (red); column 3, merged GFP and RFP signals; column 4, merged GFP and RFP signals with chlorophyll autofluorescence (blue). Scale bars represent 5 µm.

A CNGC19_FL_-mCherry fusion was generated to directly compare the subcellular localization patterns of full-length CNGC19 and CNGC20 within the same cell. Protoplasts co-transfected with CNGC19_FL_-mCherry and CNGC20_FL_-GFP displayed completely co-localizing patterns of GFP and RFP fluorescence (Fig. 8). In addition, the presence of the frequent punctate structures derived from CNGC20_FL_-GFP was greatly diminished when it was co-expressed with CNGC19_FL_-mCherry.

### Punctate CNGC20_N2_-GFP and CNGC20_FL_-GFP signals co-localize with the Golgi marker Man49-mCherry

**Figure 9.**
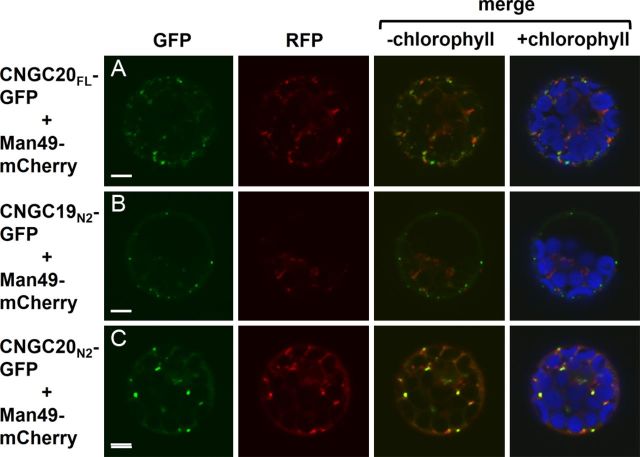
Comparison of CNGC19_N2_-GFP and CNGC20_N2_-GFP with a marker for Golgi. Confocal laser scanning microscope images of leaf protoplasts co-transfected with the Golgi marker Man49-mCherry and (A) CNGC20_FL_-GFP, (B) CNGC19_N2_-GFP or (C) CNGC20_N2_-GFP. Column 1, GFP signal (green); column 2, RFP signal (red); column 3, merged GFP and RFP signals; column 4, merged GFP and RFP signals with chlorophyll autofluorescence (blue). Scale bars represent 5 µm.

Co-transfection experiments with additional organelle markers were performed to investigate the punctate labelling pattern of CNGC20_FL_-GFP. The CNGC20_FL_-GFP punctate signals did not overlap with BiP1-mCherry-HDEL **[****Supporting Information****]**, an endoplasmic reticulum (ER) marker generated by fusing *A. thaliana* ER luminal binding protein 1 (BiP1) to a modified version of mCherry harbouring the C-terminal ER retention motif, HDEL. We also did not observe co-localization between CNGC20_FL_-GFP and markers for the mitochondria **[Supporting Information]** or peroxisomes **[****Supporting Information****]**. However, co-expression with the Golgi marker Man49-mCherry (Nelson *et al.* 2007) revealed that some (but not all) of the punctate CNGC20_FL_-GFP signals overlapped with Man49-mCherry fluorescence (Fig. 9A).

The punctate distribution patterns of partial fusion constructs, CNGC19_N2_-GFP and CNGC20_N2_-GFP, were also compared with the Golgi marker. Interestingly, whereas CNGC19_N2_-GFP did not co-localize with Man49-mCherry (Fig. 9B), CNGC20_N2_-GFP fluorescence overlapped with the Golgi marker (Fig. 9C). Not all of the punctate structures labelled by the Golgi marker were co-labelled by CNGC20_N2_-GFP (Fig. 9C), possibly indicating that CNGC20_N2_-GFP is only targeted to, or accumulates within, a subset of Golgi.

### Localization of native CNGC20 at the membrane of vacuoles by immunoelectron microscopy

**Figure 10.**
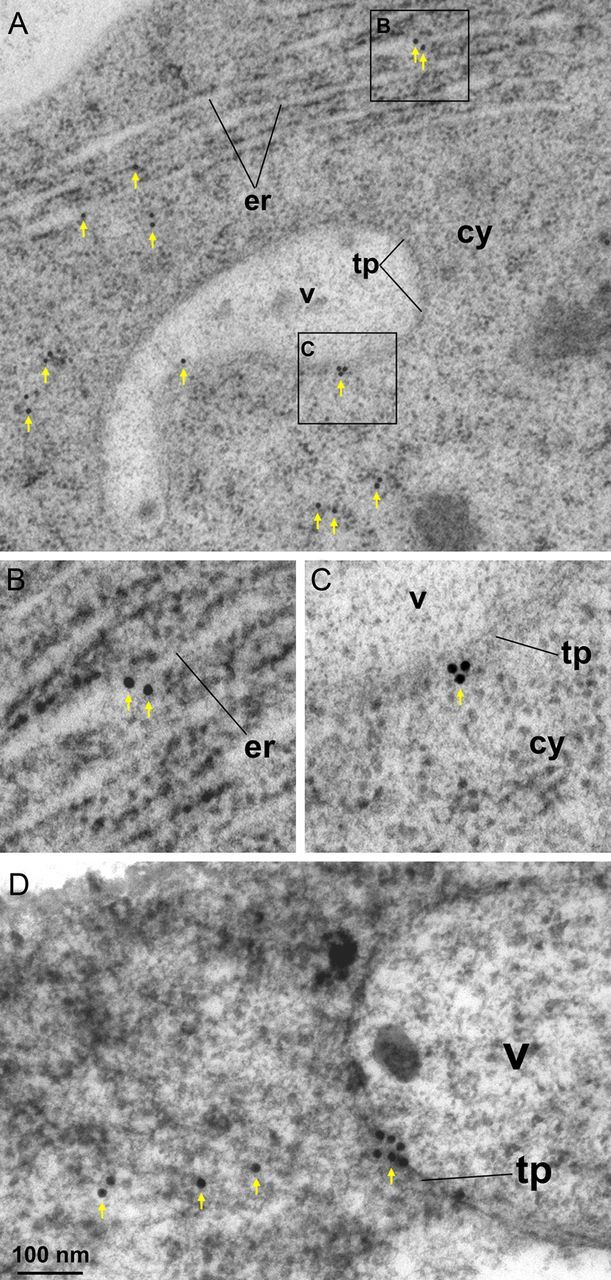
Detection of CNGC20 at vacuolar membranes by immunoelectron microscopy. (A, D) Transverse ultrathin sections of *Arabidopsis* root tips were labelled with anti-AtCNGC20 antiserum and then examined by TEM. (B, C) Higher magnification of the boxed regions in (A). cy, cytoplasm; er, endoplasmic reticulum; v, vacuole; tp, tonoplast. A 15 nm gold–anti-rabbit secondary antibody is used in all labelling.

To verify that CNGC20 is targeted to vacuolar membranes, immunolocalization experiments were performed using an affinity-purified rabbit polyclonal antiserum raised against a peptide unique to CNGC20. *Arabidopsis* root sections immunolabelled with the CNGC20 antibody were examined by transmission electron microscopy (TEM). Anti-CNGC20 labelling was observed at the membranes of vacuoles (Fig. 10A, C and D). Labelling was also detected at the ER (Fig. 10A and B), and on darker-stained areas within the cytoplasm (Fig. 10A and D), which may represent CNGC20 at various stages along the secretory pathway. We did not detect labelling in control samples challenged with the rabbit pre-immune serum in place of the anti-CNGC20 antibody [**Supporting Information****]** or in leaf sections containing chloroplasts incubated with the anti-CNGC20 antibody **[Supporting Information]**. Unlike the over-expression that occurs when CNGC20_FL_-GFP is transiently expressed in protoplasts under the CaMV 35S promoter, immunolocalization allows for the detection of CNGC20 under its normal expression pattern in plants. Thus, immunoelectron microscopy confirmed the presence of native CNGC20 *in planta* at the membrane of vacuoles.

## Discussion

In *Arabidopsis*, the CNGC phylogenetic tree consists of 20 members, of which CNGC19 and CNGC20 are the sole members of subgroup IV-A. They are distinguished among CNGCs by their long and novel conserved N-termini, yet retain key features common to all CNGCs such as the overlapping cyclic nucleotide- and calmodulin-binding domains of the C-terminus, six predicted membrane spanning domains and a close resemblance to Shaker-type K^+^ channels. To better understand the roles that CNGC19 and CNGC20 play in regulating cation fluxes in plants, we investigated their subcellular locations using the leaf mesophyll protoplast transient expression system. Although the conserved hydrophilic N-termini of CNGC19 and CNGC20 are predicted to contain chloroplast transit peptides, translational fusions of GFP with CNGC19 or CNGC20 did not localize to chloroplasts. We instead found that CNGC19_FL_-GFP and CNGC20_FL_-GFP co-localized with markers for the vacuole membrane (αTIP-mCherry; γTIP-mCherry) when simultaneously expressed in protoplasts.

Under our experimental conditions, αTIP- and γTIP-mCherry typically did not appear to label the limiting membrane of the large central vacuole. This is consistent with previous studies showing that epitope-tagged or fluorescent protein-tagged versions of αTIP, δTIP and γTIP strongly label relatively small elliptical structures when transiently expressed in protoplasts or cultured cells (Park *et al.* 2004; Kim *et al.* 2006; Saito *et al.* 2011). These structures may represent either small vacuoles or ring-like extensions of the central vacuole membrane that are referred to as vacuole ‘bulbs’. Vacuolar bulbs were first identified in *Arabidopsis* transgenic plants stably expressing γTIP-GFP under the CaMV 35S promoter (Saito *et al.* 2002). The intensity of γTIP-GFP fluorescence in bulbs is ∼3-fold higher than that in the limiting membrane of the central vacuole (Saito *et al.* 2002), and their formation is correlated with the fusion of small vacuoles (Saito *et al.* 2011). It is speculated that vacuolar bulbs may serve as a reservoir of membranes to facilitate the rapid expansion or transformation of vacuoles or plant response to salt stress (Saito *et al*. 2002; Boursiac *et al.* 2005), or as specialized subregions of the vacuole where hydrolytic activities are localized (Saito *et al.* 2002; Zheng and Staehelin 2011). In addition to labelling bulb-like structures, CNGC19_FL_-GFP and CNGC20_FL_-GFP occasionally co-localized with short, non-elliptical membranes of intense vacuole marker fluorescence. We hypothesize that these co-labelled membranes correspond to regions of the tonoplast where vacuole bulbs were unfolded to expand the limiting membrane of the central vacuole.

Protoplasts expressing CNGC20_FL_-GFP frequently exhibited punctate GFP fluorescence that partially co-localized with the Golgi marker Man49-mCherry. Thus, CNGC20_FL_-GFP may have a lower efficiency in trafficking to the vacuole than CNGC19_FL_-GFP under our experimental conditions, resulting in the accumulation of CNGC20_FL_-GFP in Golgi and possibly other undefined punctate subcellular structures. We observed a decrease in punctate labelling when CNGC20_N2_-GFP was co-expressed with CNGC19_FL_-mCherry, with 70–80 % more of the cells exhibiting some vacuolar labelling, possibly indicating an increased efficiency in CNGC20_FL_-GFP trafficking to the vacuolar membrane in the presence of co-expressed CNGC19_FL_-mCherry. Since plant CNGC polypeptides most likely assemble as tetramers to form functional cation channels (Hua *et al.* 2003), we speculate that the simultaneous expression of CNGC19_FL_-mCherry and CNGC20_FL_-GFP causes the chimeric proteins interacting with each other to form CNGC19/CNGC20 hetero-multimers, which are transported together to the vacuole membrane.

Protoplasts expressing GFP fused to the entire hydrophilic N-terminal region of CNGC19 or CNGC20 (CNGC19_N2_-GFP; CNGC20_N2_-GFP) do not display diffuse cytosolic labelling, but instead exhibit punctate labelling. This suggests that the N-terminal regions of CNGC19 and CNGC20 contain information that influences protein sorting, but are not sufficient to direct proteins to the vacuole. Interestingly, the N-terminal regions of group IV-A CNGCs contain a novel 19-aa conserved motif (LxxSGxLGxCxDPxCxxCP). Since GFP fusions harbouring shortened versions of the N-terminal region truncated just prior to this motif (CNGC19_N1_-GFP; CNGC20_N1_-GFP) exhibit diffuse labelling of the cytosol, we speculate that the 19-aa interval may play a role in protein sorting. Interestingly, in protoplast co-transfection experiments, the Golgi marker Man49-mCherry co-localized with CNGC20_N2_-GFP, but not with CNGC19_N2_-GFP. This suggests that differences exist between the protein sorting information contained within the N-terminal hydrophilic regions of CNGC19 and CNGC20. These differences may contribute, at least in part, to the dissimilar localization patterns of our full-length CNGC19_FL_-GFP and CNGC20_FL_-GFP fusions with regard to the absence or presence of punctate labelling (respectively).

The presence of CNGC19 and CNGC20 at vacuolar membranes suggests that these channels serve as pathways for the passive transport of cations between the vacuole and cytosol. Since the genes encoding CNGC19 and CNGC20 are upregulated by salt stress (Maathuis, 2006; Kugler *et al.* 2009; Yuen and Christopher 2010), one possible function of these channel proteins is to ameliorate the effects of deleterious levels of Na^+^ in the cytosol by facilitating Na^+^ redistribution between the cytosol and vacuole. It is unlikely, however, that CNGC19 and CNGC20 play a direct role in the sequestration of Na^+^ to the central vacuole since Na^+^ must be actively transported against its electrochemical gradient, a function performed by vacuolar Na^+^/H^+^ (NHX) antiporters (Apse *et al.* 1999, 2003). An alternative possibility is that CNGC19 and CNGC20 facilitate the plant's response to salinity by mediating Ca^2+^ signalling. Salt stress induces a rapid rise in cGMP levels, and a transient increase in free cytosolic [Ca^2+^]; at moderate salt concentrations (50 mM), suppressing cGMP accumulation with an inhibitor of guanylyl cyclases also diminishes the Ca^2+^ spike (Donaldson *et al.* 2004). Cyclic nucleotide-gated channels, which are activated by cyclic nucleotides (Li *et al.* 2005), could function as a link between cGMP accumulation and the influx of Ca^2+^ into the cytosol (Donaldson *et al.* 2004). The potential involvement of vacuolar CNGCs in Ca^2+^ signalling is circumstantially supported by experiments in tobacco protoplasts that have demonstrated that cAMP and cGMP can trigger the influx of Ca^2+^ to the cytosol from both intracellular and extracellular Ca^2+^ stores (Volotovski *et al.* 1998). In addition to being upregulated by salt stress, *CNGC19* and *CNGC20* have been implicated in the *Arabidopsis* response to infection by bacterial and fungal pathogens (Moeder *et al.* 2011). If CNGC19 and CNGC20 are indeed Ca^2+^ channels, they may serve a similar molecular function in the response to both abiotic and biotic stress by mediating calcium signalling through the release of vacuolar Ca^2+^ into the cytosol.

## Conclusions

CNGC19 and CNGC20 are components of vacuole membranes. Under the protoplast transient expression system, CNGC20 is weakly trafficked to the vacuole.

However, co-expression of CNGC19 and CNGC20 results in efficient transport of CNGC20 to the vacuolar membrane, possibly due to the formation of heteromultimeric channels. How CNGC19 and CNGC20 influence cation fluxes within plant cells in response to salt stress and biotic stress remains unclear. Future experiments defining the permeability of these channels to various cations will clarify whether CNGC19 and CNGC20 are directly involved in the subcellular redistribution of Na^+^ or function as mediators of Ca^2+^ signalling.

## Sources of Funding

This research was supported by a Hatch project award (HAW00507-H) and the Advances in Biosciences Education programme funded by the National Science Foundation (grant no. MCB-09-58107).

## Contributions by the Authors

D.A.C. conceived the project, and D.A.C. and C.C.Y.Y. designed the protoplast transient expression experiments and developed all the new fusion constructs utilized in this study. D.A.C. performed all the immunoelectron microscopy experiments. C.C.Y.Y. conducted protoplast transfections and analysed the samples through confocal microscopy. D.A.C. and C.C.Y.Y. wrote the manuscript, and read and approved the final version for submission.

## Conflict of Interest Statement

None declared.

## Supporting Information

The following Supporting Information is available in the online version of this article.

**File 1**: Figure. Comparison of CNGC19_FL_- and CNGC20_FL_-GFP localization with a marker for the ER. Confocal laser scanning microscope images of leaf protoplasts co-transfected with the ER marker BiP1-mCherry-HDEL and (A) CNGC19_FL_-GFP or (B) CNGC20_FL_-GFP. Column 1, GFP signal (green); column 2, RFP signal (red); column 3, merged GFP and RFP signals; column 4, merged GFP and RFP signals with chlorophyll autofluorescence (blue). Scale bars represent 5 µm.

**File 2**: Figure. Comparison of CNGC19_FL_- and CNGC20_FL_-GFP localization with a marker for mitochondria. Confocal microscopy images of leaf protoplasts transiently expressing (A) CNGC19_FL_-GFP or (B) CNGC20_FL_-GFP, and stained with MitoTracker Orange. Column 1, GFP signal (green); column 2, MitoTracker Orange signal (red); column 3, merged GFP and MitoTracker Orange signals; column 4, merged GFP and MitoTracker Orange signals with chlorophyll autofluorescence (blue). Scale bars represent 5 µm.

**File 3**: Figure. Comparison of CNGC19_N2_- and CNGC20_N2_-GFP localization with a marker for peroxisomes. Confocal laser scanning microscope images of leaf protoplasts co-transfected with the peroxisome marker mCherry-SKL and (A) CNGC19_N2_-GFP or (B) CNGC20_N2_-GFP. Column 1, GFP signal (green); column 2, RFP signal (red); column 3, merged GFP and RFP signals; column 4, merged GFP and RFP signals with chlorophyll autofluorescence (blue). Scale bars represent 5 µm.

**File 4**: Figure. Pre-immune serum staining of roots. Immunolabelling of *Arabidopsis* cryo-fixed thin root tissue sections with rabbit pre-immmune serum and anti-rabbit 15 nm gold-conjugated secondary antiserum as a negative control. Samples were viewed via TEM as described in the Methods. No labelling was observed. G, Golgi apparatus, Cy, cytoplasm; Vc, vacuole; ER, rough endoplasmic reticulum; Pm, plasma membrane; M, mitochondrion.

**File 5**: Figure. Immunolabelling of leaves. Immunolabelling of *Arabidopsis* cryo-fixed thin leaf tissue sections with anti-CNGC20 antiserum and anti-rabbit 15 nm gold-conjugated secondary antiserum. Samples were viewed via TEM as described in the Methods. No labelling was observed in the chloroplasts (CT); some labelling was observed in the edges of vacuoles (V).

**File 6**: Figure. Immunoblot analysis of CNGC20 in *Arabidopsis* total cellular proteins. Immunoblot analysis was performed using the anti-CNGC20-specific peptide antiserum on 40 μg of total seedling proteins (14-day-old) from wild type (WT) and the CNGC20 T-DNA mutant (MUT). The homozygous T-DNA insert (SALK_129133.22.05) is in the fourth exon of the CNGC20 locus (At3g17700). The antiserum detects a single band of ∼84 kDa in the wild-type protein sample, which is the predicted size of CNGC20, whereas no CNGC20 protein is detected in the mutant. This indicates that the antiserum is specific to CNGC20. Coomassie-stained proteins (COOM) are shown from a duplicate gel.

Additional Information
