# Supplementary material for: The group IV-A cyclic nucleotide-gated channels, CNGC19 and CNGC20, localize to the vacuole membrane in Arabidopsis thaliana
Source: AoB Plants. 2013 Feb 22;5:plt012. doi: 10.1093/aobpla/plt012 (PMC4455320; doi:10.1093/aobpla/plt012)
Supplement: Additional Information [file supp_5_plt012_index.html]

The group IV-A cyclic nucleotide-gated channels, CNGC19 and CNGC20, localize to the vacuole membrane in Arabidopsis thaliana — Additional Information 

# The group IV-A cyclic nucleotide-gated channels, CNGC19 and CNGC20, localize to the vacuole membrane in *Arabidopsis thaliana*

## Additional Information

**Files in this Data Supplement:**

- Additional Information - doc file
- Additional Information - tif file
- Additional Information - tif file
- Additional Information - tif file
- Additional Information - tif file
- Additional Information - tif file
- Additional Information - tif file
