# Supplementary material for: The group IV-A cyclic nucleotide-gated channels, CNGC19 and CNGC20, localize to the vacuole membrane in Arabidopsis thaliana
Source: AoB Plants. 2013 Feb 22;5:plt012. doi: 10.1093/aobpla/plt012 (PMC4455320; doi:10.1093/aobpla/plt012)
Supplement: Additional Information [file supp_plt012_pls012supp.doc]

**ADDITIONAL INFORMATION**

The following **[ADDITIONAL INFORMATION**] is available in the on-line version of this article -

**File 1.** Figure. Comparison of CNGC19_FL_- and CNGC20_FL_-GFP localization with a marker for the ER. Confocal laser scanning microscope images of leaf protoplasts cotransfected with the ER marker BiP1-mCherry-HDEL and (A) CNGC19_FL_-GFP, or (B) CNGC20_FL_-GFP. Column 1, GFP signal (green); column 2, RFP signal (red); column 3, merged GFP and RFP signals; column 4, merged GFP and RFP signals with chlorophyll autofluorescence (blue). Scale bars represent 5 µm.

**File 2.** Figure. Comparison of CNGC19_FL_- and CNGC20_FL_-GFP localization with a marker for mitochondria. Confocal microscopy images of leaf protoplasts transiently expressing (A) CNGC19_FL_-GFP, or (B) CNGC20_FL_-GFP, and stained with MitoTracker Orange. Column 1, GFP signal (green); column 2, MitoTracker Orange signal (red); column 3, merged GFP and MitoTracker Orange signals; column 4, merged GFP and MitoTracker Orange signals with chlorophyll autofluorescence (blue). Scale bars represent 5 µm.

**File 3.** Figure. Comparison of CNGC19_N2_- and CNGC20_N2_-GFP localization with a marker for peroxisomes. Confocal laser scanning microscope images of leaf protoplasts cotransfected with the peroxisome marker mCherry-SKL and (A) CNGC19_N2_-GFP, or (B) CNGC20_N2_-GFP. Column 1, GFP signal (green); column 2, RFP signal (red); column 3, merged GFP and RFP signals; column 4, merged GFP and RFP signals with chlorophyll autofluorescence (blue). Scale bars represent 5 µm.

**File 4.** Figure. Pre-immune serum staining of roots. Immunolabeling of Arabidopsis cryo-fixed thin root tissue sections with rabbit pre-immmune serum and anti-rabbit 15 nm gold-conjugated secondary antiserum as a negative control. Samples were viewed via TEM as described in the Materials and Methods. No labeling was observed. G, Golgi apparatus, Cy, Cytoplasm; Vc, vacuole; ER, rough endoplasmic reticulum; Pm, plasma membrane; M, mitochondrion.

**File 5.** Figure. Immunolabelling of leaves. Immunolabelling of Arabidopsis cryo-fixed thin leaf tissue sections with anti-CNGC20 antiserum and anti-rabbit 15 nm gold-conjugated secondary antiserum. Samples were viewed via TEM as described in the Materials and Methods. No labelling was observed in the chloroplasts (CT) some labelling was observed in edge of vacuoles (V).

**File 6.** Figure. Immunoblot analysis of CNGC20 in Arabidopsis total cellular proteins. Immunoblot analysis was performed using the anti-CNGC20-speciifc peptide antiserum on 40 μg total seedling proteins (14 day-old) from wild type (WT) and the CNGC20 T-DNA mutant (MUT). The homozygous T-DNA insert (SALK_129133.22.05) is in the forth exon of the CNGC20 locus (At3g17700). The antiserum detects a single band of approximately 84 kD in the wild type protein sample, which is the predicted size of CNGC20, whereas no CNGC20 protein is detected in the mutant. This indicates the antiserum is specific to CNGC20. Coomassie stained proteins (COOM) shown from a duplicate gel.
