# Supplementary figures and images for: The group IV-A cyclic nucleotide-gated channels, CNGC19 and CNGC20, localize to the vacuole membrane in Arabidopsis thaliana
Source: AoB Plants. 2013 Feb 22;5:plt012. doi: 10.1093/aobpla/plt012 (PMC4455320; doi:10.1093/aobpla/plt012)

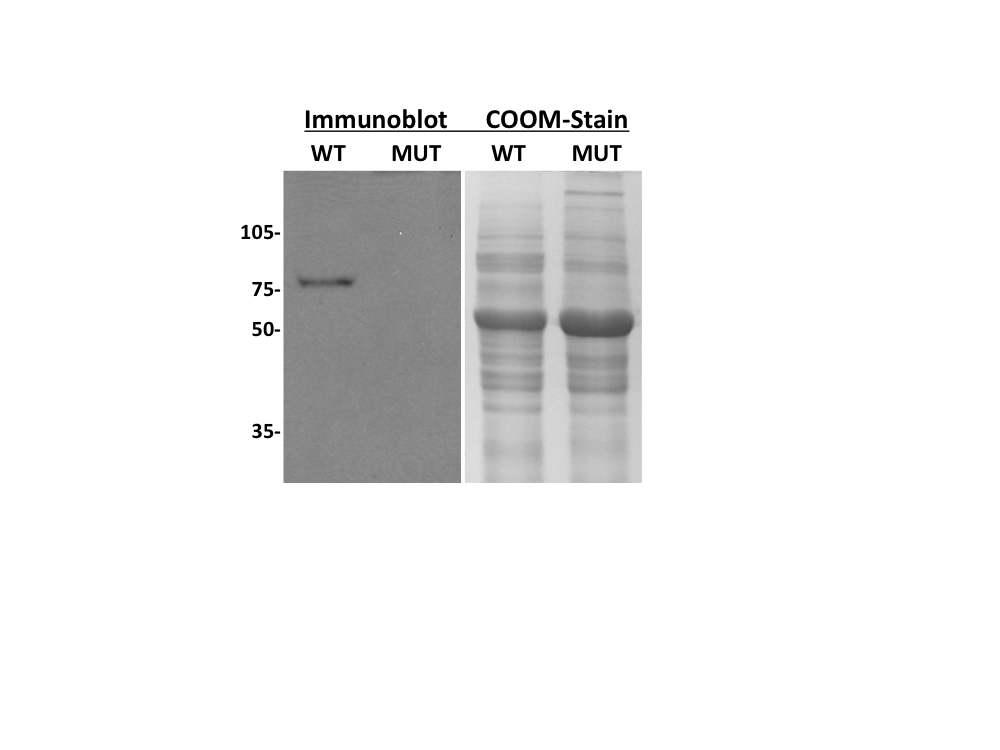

Supplement: Additional Information [file supp_plt012_plt012supp_fig1.tif]

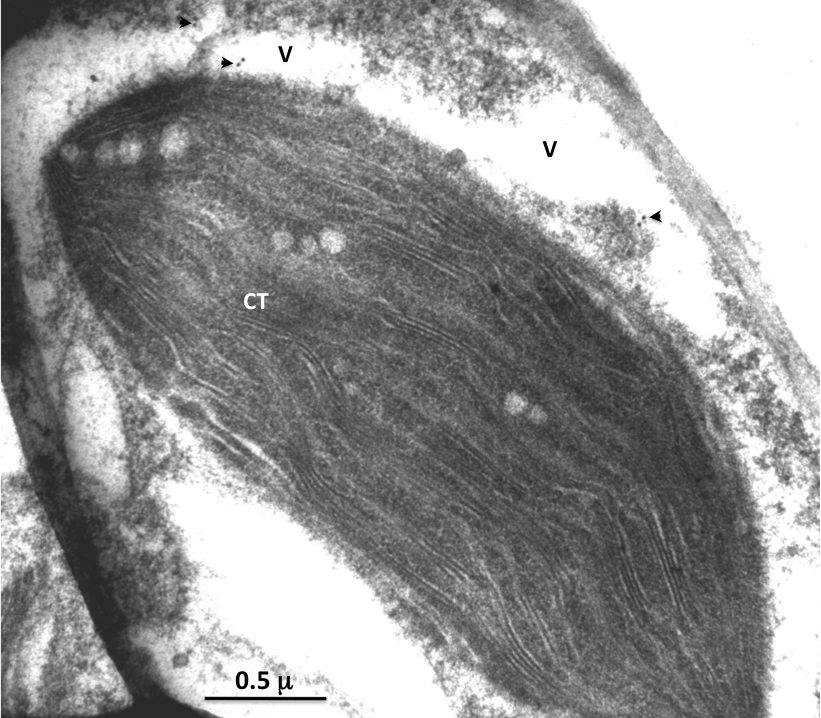

Supplement: Additional Information [file supp_plt012_plt012supp_fig2.tif]

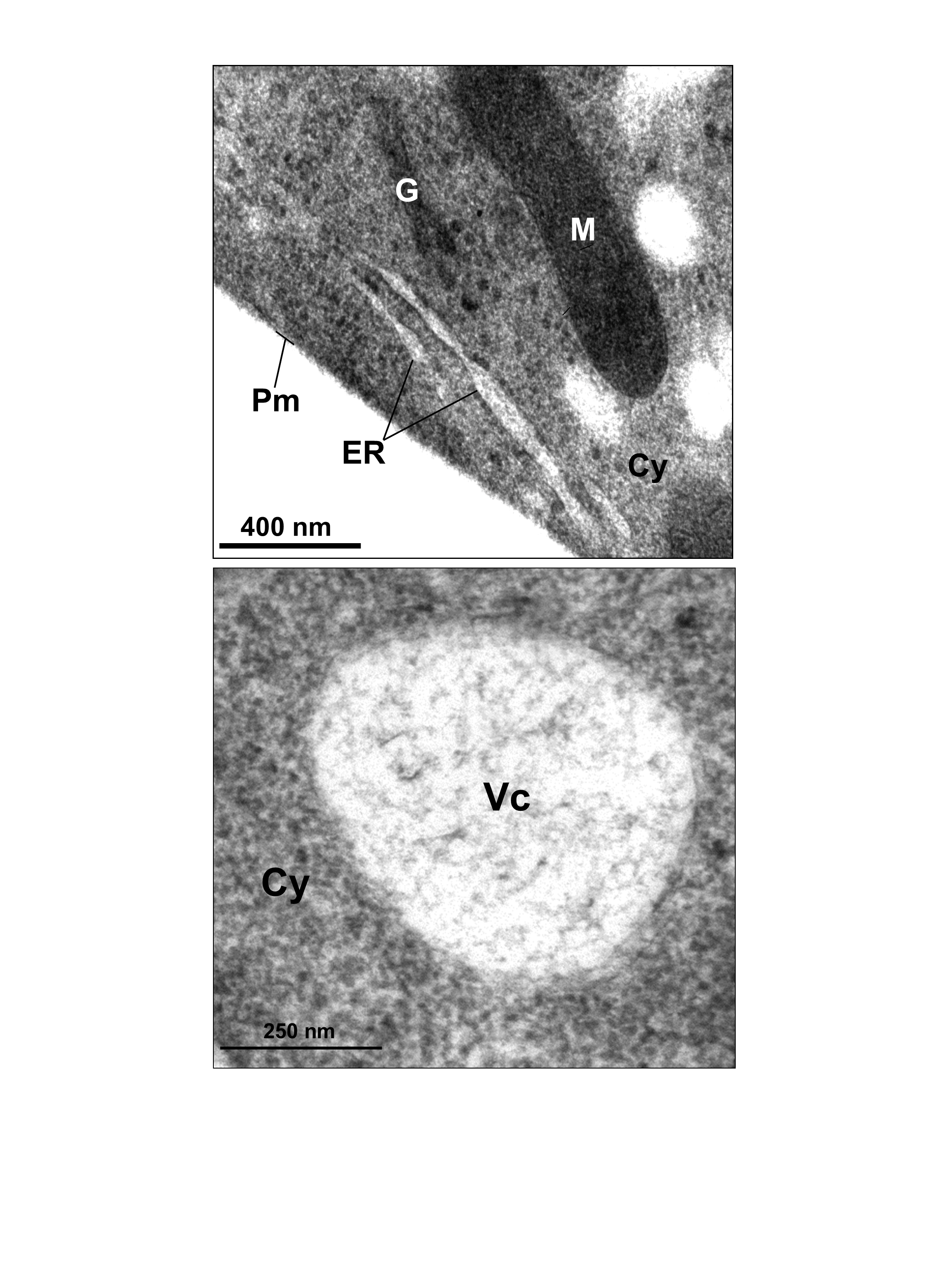

Supplement: Additional Information [file supp_plt012_plt012supp_fig3.tif]

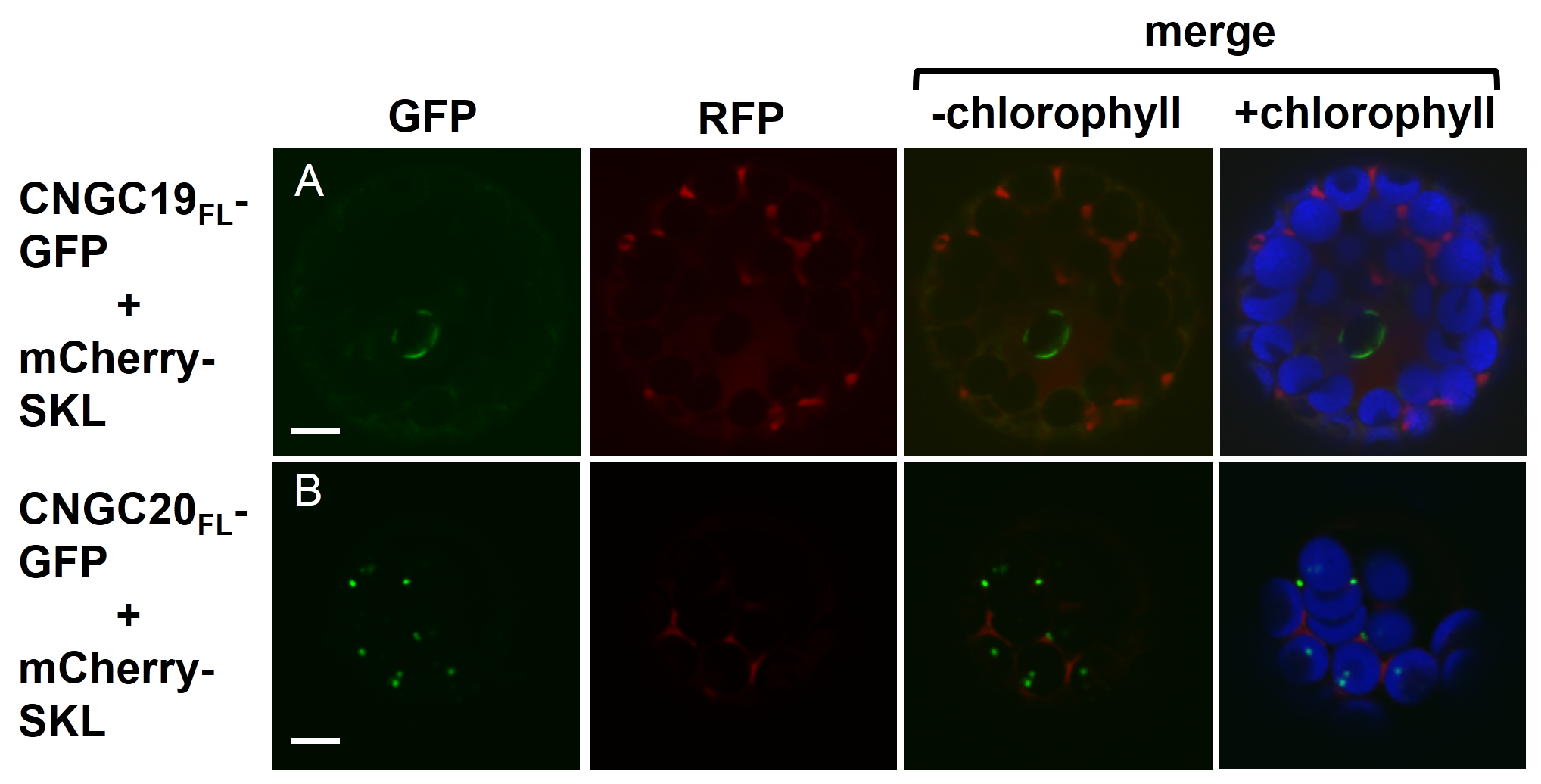

Supplement: Additional Information [file supp_plt012_plt012supp_fig4.tif]

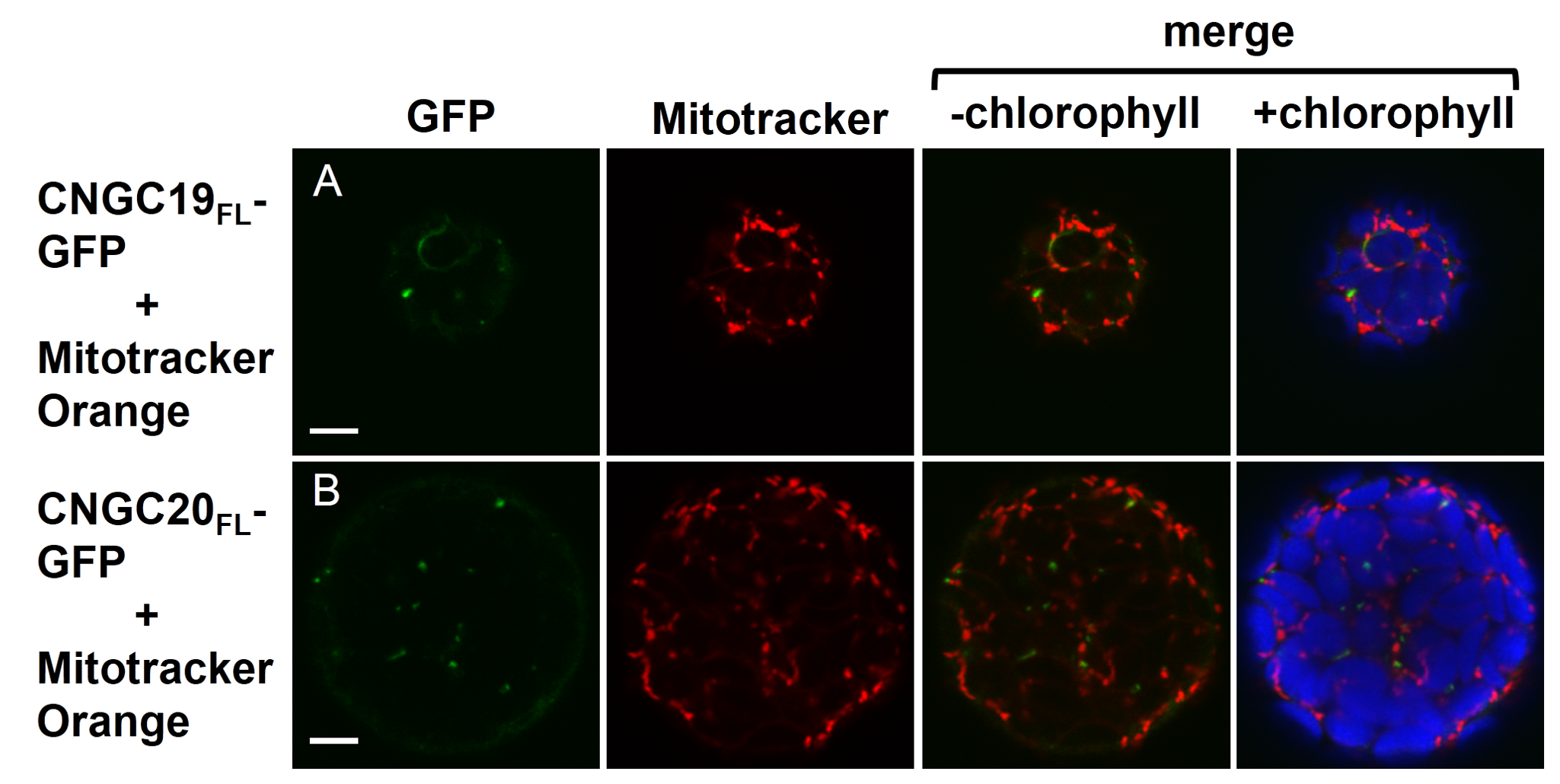

Supplement: Additional Information [file supp_plt012_plt012supp_fig5.tif]

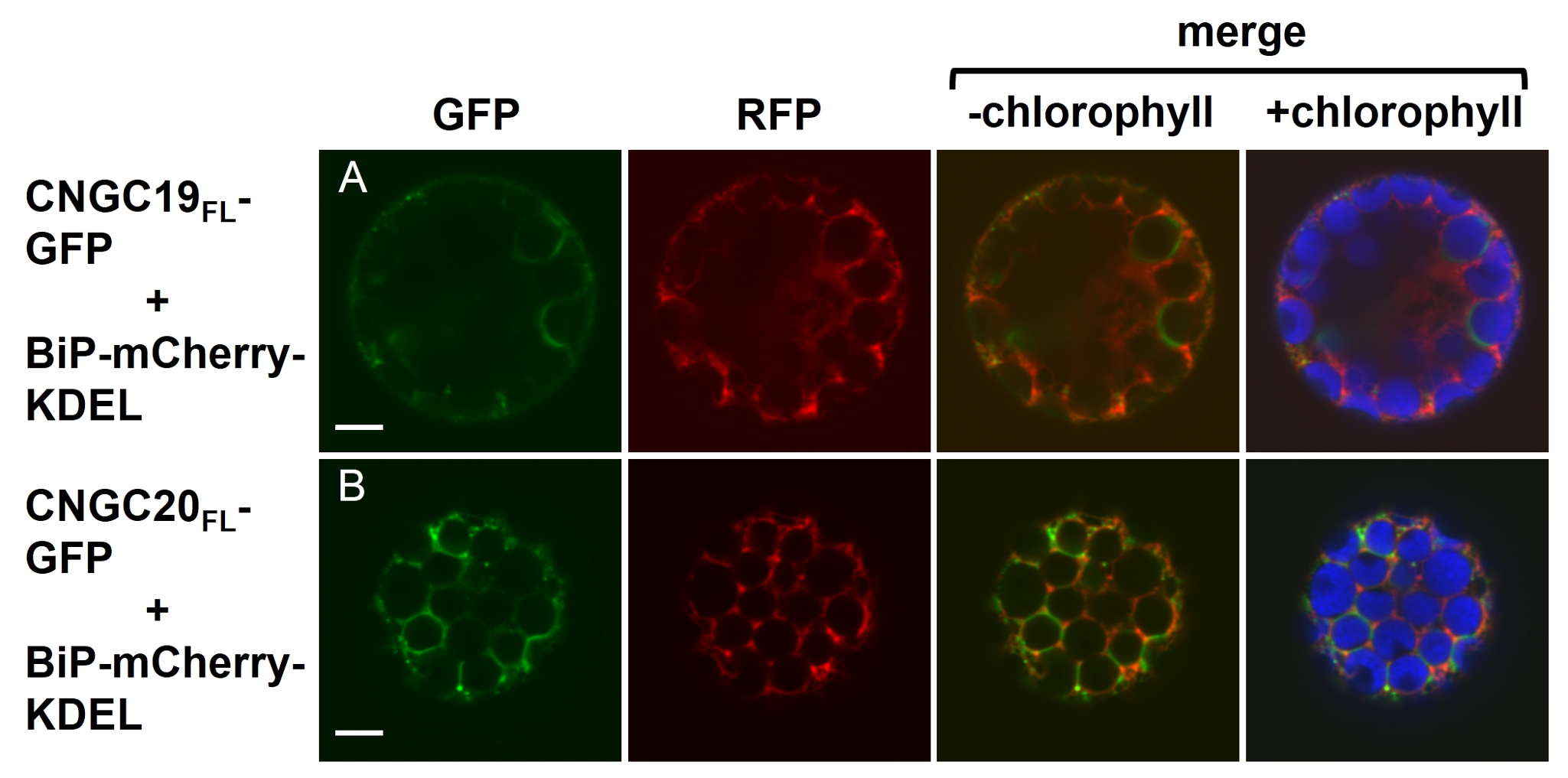

Supplement: Additional Information [file supp_plt012_plt012supp_fig6.tif]
